# Colovesical Fistula Surgically Managed With Delayed Computed Tomography Detection

**DOI:** 10.7759/cureus.74277

**Published:** 2024-11-22

**Authors:** Abraham Karimi, Jay Xiong, Jasleen Kaur

**Affiliations:** 1 Medical School, St. Joseph’s Medical Center, Stockton, USA; 2 Medical School, Touro College of Osteopathic Medicine, Vallejo, USA; 3 Internal Medicine, St. Joseph’s Medical Center, Stockton, USA

**Keywords:** colovesical fistula, ct, enterocutaneous fistula, fecaluria, peritoneal abscess

## Abstract

Colovesical fistula is the result of a pathologic connection between the bladder and colon. It can be a deadly sequela of a variety of conditions. Diagnosis is usually confirmed by a computed tomography (CT) scan of the abdomen. We present a case of a patient who had multiple CT scans that did not show signs of fistula and was still successfully treated. Clinicians should follow clinical signs for colovesical fistula and pursue treatment regardless of CT scan findings.

## Introduction

Colovesical fistula is an abnormal connection between the bladder and colon. The most common causes that result in this connection include diverticular disease, malignancy, and Crohn’s disease. Other less common causes include iatrogenic injury from surgeries, tuberculosis, and pelvic radiation therapy [1-3]. Common symptoms that patients with colovesical fistula may present include pneumaturia, urinary tract infection with dysuria and polyuria, fecaluria, hematuria, and orchitis [4]. Other causes of some of the pathognomonic signs of colovesical fistula include emphysematous cystitis with gas-forming organisms or recent bladder instrumentation. Diagnosis of a fistula is commonly done clinically but imaging is useful as it helps with identifying the location of the fistula, spotting any abnormal anatomy, and discovering tumors. Imaging is routinely done with a computed tomography (CT) scan with oral (PO) or rectal (PR) contrast and without intravenous (IV) contrast [5,6]. As for treatment, the course depends on if the patient is a surgical candidate. However, some research suggests that surgery may not always be the answer and that conservative management leads to similar outcomes in benign colovesical fistula [7]. In this study, we report a case of a male with fecaluria, sepsis, and pelvic abscess. He was found to have a colovesical fistula on day 12 of admission after multiple prior CT scans from the same admission did not show the fistula. However, he was still successfully managed to a stable condition with a multidisciplinary team of multiple specialists who treated the patient with intraperitoneal drainage, antibiotics, and nephrostomy tube placement. The patient ultimately left against medical advice before completing his antibiotic treatment and returned a few days later, where he did eventually have surgical treatment.

## Case presentation

A 65-year-old male with a medical history of chronic kidney disease, extensive intraperitoneal surgical history, and multiple enteric fistulas that likely resulted from his surgical history was brought in by ambulance from his clinic visit for hypotension. He originally went to his clinic visit for complaints of dysuria and thick brown discharge in his urine for the past three days. He was found to have a blood pressure of 76/34 mmHg and was sent to the emergency department (ED) due to concern for septic shock. Relevant past medical history includes being admitted in the past few months and undergoing multiple drainages of abscess, right nephroureteral tube placement, and robotic right hemicolectomy and resection of the terminal ileum with primary ileocolic anastomosis two weeks prior.

On presentation in the ED, the patient was complaining of generalized abdominal pain. Vital signs were significant for blood pressure of 85/44 mmHg. On physical examination, the patient was lethargic, difficult to arouse, minimally interactive, and alert and oriented x 1-2 - able to state his name and was aware he was at the hospital. His abdomen was soft, non-distended, and with no guarding or rebound. There were abdominal drains in place. Labs on the date of admission showed a complete blood count as follows: white blood cell count of 13.5 thousand/uL, hemoglobin of 7.4 g/dL, hematocrit of 22.3%, and platelet count of 662 thousand/uL. Blood, urine, and abscess cultures were positive for *Enterobacter cloacae* complex with the urine culture additionally showing *Enterococcus faecalis*. Table 1 summarizes the patient's laboratory results. He was treated with IV vancomycin per pharmacy, IV cefepime 2000 mg daily, and IV metronidazole 500 mg every eight hours. He was on a levophed drip with a mean arterial pressure goal above 65 mmHg.

**Table 1:** Vitals and labs eGFRcr: Creatinine-based estimated glomerular filtration rate; BUN: Blood urea nitrogen; SpO_2_: Peripheral capillary oxygen saturation; CO_2_: Carbon dioxide

Vitals and Laboratory Results		
Vitals/Labs	Range	Reference Ranges
Temperature	36.7 °C	36-37.5 °C
Heart rate	96	60-99
Respiratory rate	21	13-20
Blood pressure	85/44	91-139 / 51-89
SpO_2_	96% on room air	>92%
White blood cells	13.5 thousand/uL	4.0-10.0 thousand/uL
Hemoglobin	7.4 gm/dL	13.6-17.0 gm/dL
Hematocrit	22.30%	39-49%
Platelets	662 thousand/uL	150-400 thousand/uL
Sodium	128 mmol/L	136-145 mmol/L
Potassium	4.5 mmol/L	3.5-5.1 mmol/L
Chloride	97 mmol/L	98-107 mmol/L
CO_2_	20 mmol/L	22-29 mmol/L
Anion gap	11 mmol/L	5-14 mmol/L
Glucose	85 mg/dL	70-105 mg/dL
BUN	47.9 mg/dL	8.4-25.7 mg/dL
Creatinine	2.8 mg/dL	0.7-1.3 mg/dL
eGFRcr	24 mL/min	>90 mL/min
Calcium	8.5 mg/dL	8.4-10.5 mg/dL
Lactic acid	2.3 mmol/L	0.5-2.0 mmol/L
Urine culture	*Enterobacter cloacae* complex, *Enterococcus faecalis*	Negative
Blood cultures x2	*Enterobacter cloacae* complex	Negative
Abscess culture	*Enterobacter cloacae* complex, *Enterococcus faecium*, *Citrobacter amalonaticus*	Negative

**Figure 1:**
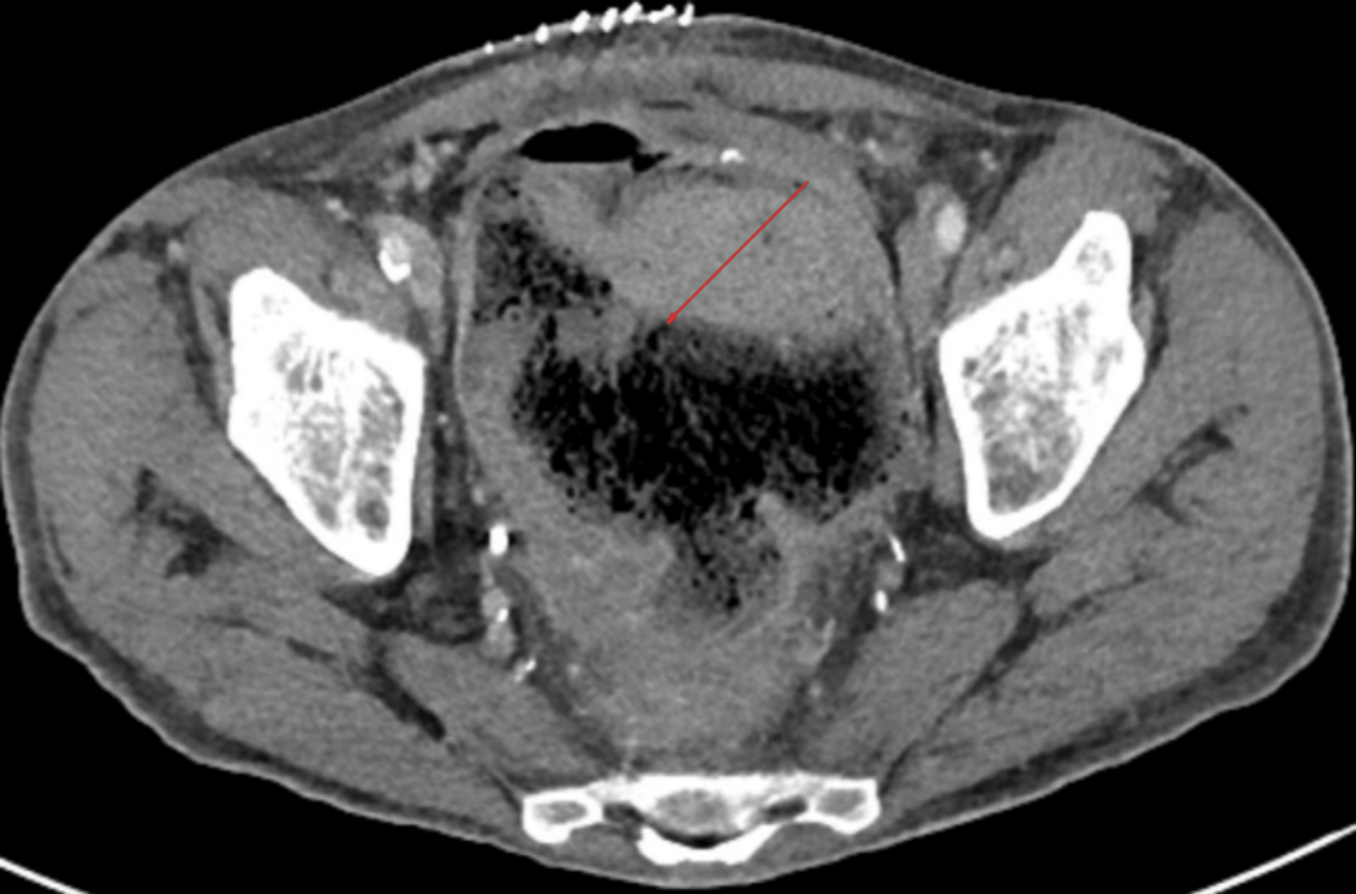
CT abdomen/pelvis in axial view demonstrating a large collection with an air-fluid level and feculent material (red arrow) in the right lower abdomen and pelvis CT: Computed tomography

**Figure 2:**
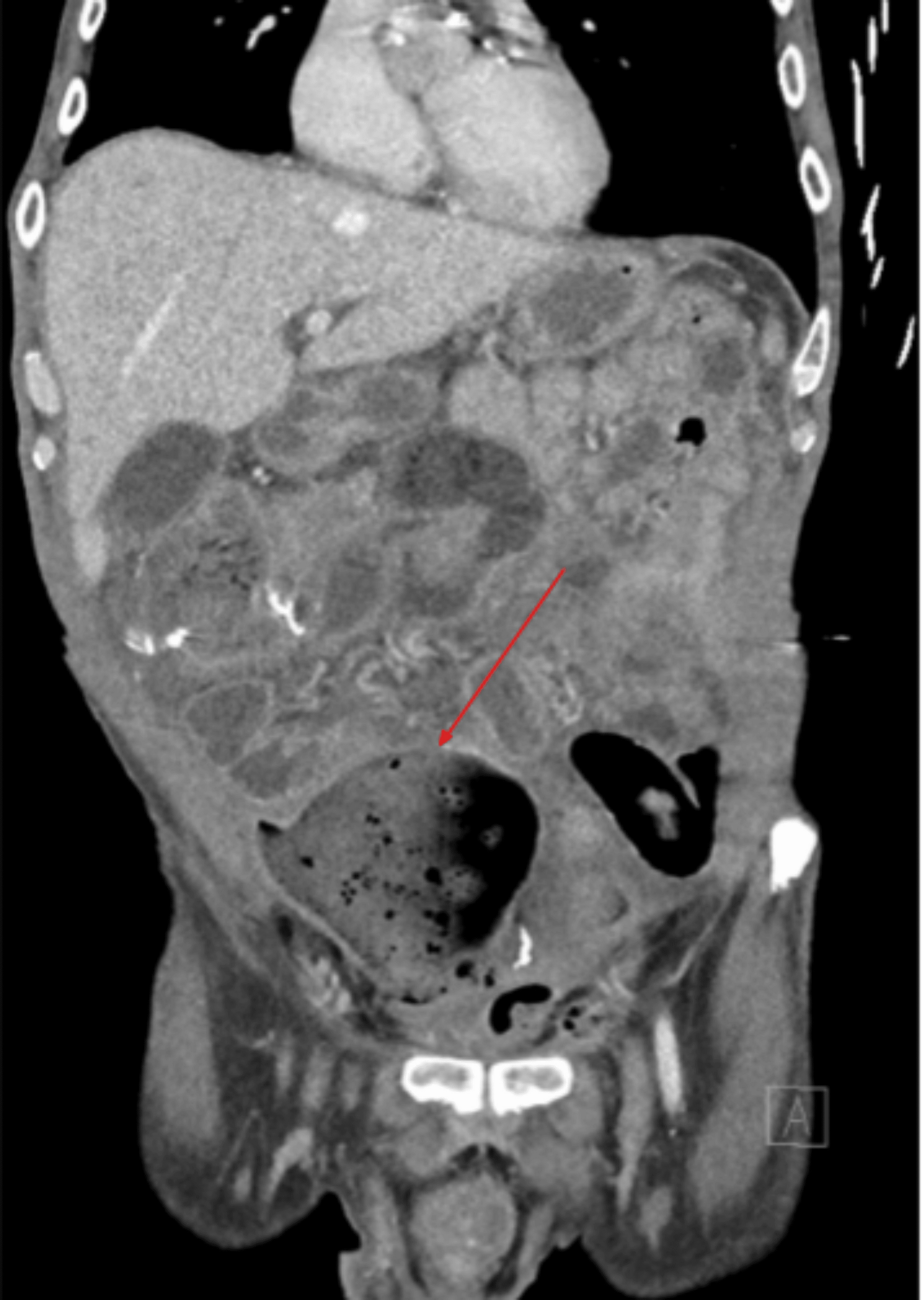
CT abdomen/pelvis in coronal view demonstrating a large collection with an air-fluid level and feculent material (red arrow) in the right lower abdomen and pelvis CT: Computed tomography

**Figure 3:**
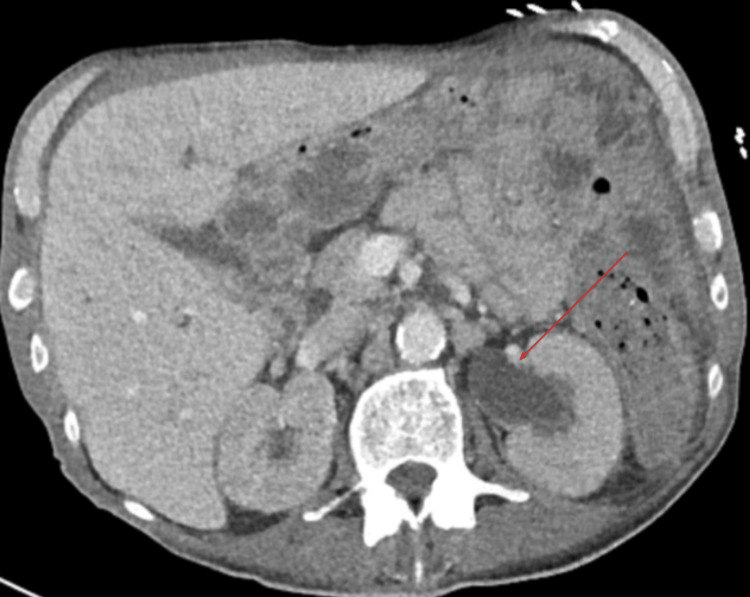
CT abdomen/pelvis in axial view demonstrating moderate to severe left hydronephrosis and hydroureter (red arrow) CT: Computed tomography

The CT scan of the abdomen and pelvis with IV contrast performed on the date of admission showed a large collection of air-fluid level and feculent material in the right lower abdomen and pelvis but was unable to optimally assess a fistulous tract to a peritoneal collection (Figures 1-2). There was also moderate to severe left hydronephrosis, and a hydroureter was seen on the CT (Figure 3).

**Figure 4:**
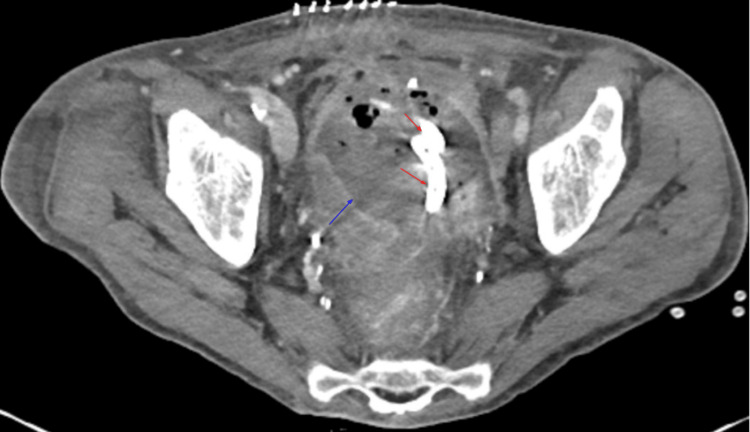
CT abdomen/pelvis in axial view demonstrating decreased size of an associated complex gas and fluid collection with feculent material (blue arrow) and two large caliber drainage catheters in place (red arrows) CT: Computed tomography

General Surgery and Urology were consulted; however, they recommended that the patient be medically managed instead as the patient was too unstable for surgery. Additionally, the patient was in the two-week postoperative period from his last recent surgery. Per the surgery team, further surgical intervention could be risky with a significant mortality rate. Per the urology team, he was also not a candidate for retrograde cystography. The patient was recommended to be only treated with IV antibiotics and drainage of collection. Interventional Radiology placed two abdominal drains into the midline abdomen and right lateral abdomen, yielding feculent fluid, and a left nephrostomy tube for urinary diversion. The following day, a CT scan of the abdomen and pelvis with IV, PO, and PR contrast was performed, and there was still no definite bladder wall disruption to suggest a fistula. Six days after the admission date, the patient was improving, and another CT scan of the abdomen and pelvis with IV contrast was performed, which showed a decreased size of the pelvic fluid but still did not demonstrate a colovesical fistula (Figure 4).

**Figure 5:**
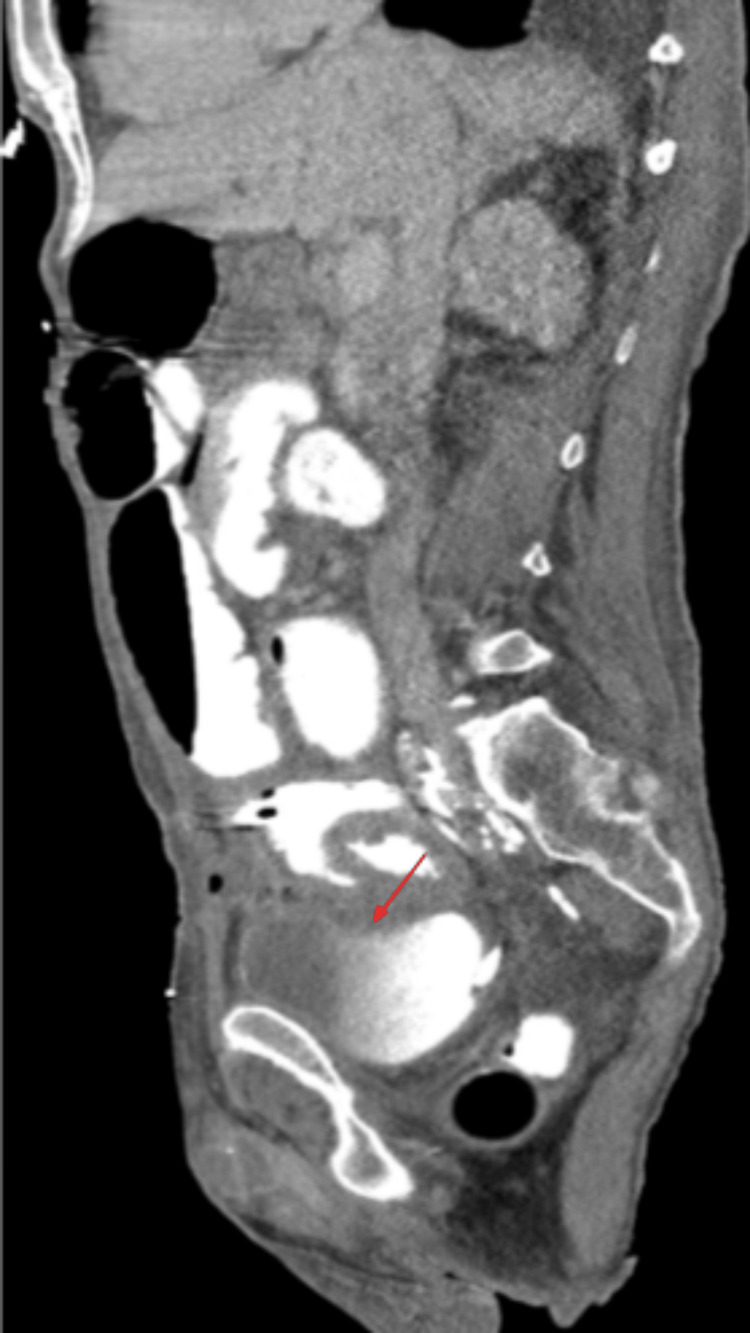
CT abdomen/pelvis in sagittal view demonstrating contrast within the bladder, which represents enterovesicular fistula (red arrow) CT: Computed tomography

A final repeat CT scan of the abdomen and pelvis with IV and PR contrast was performed to evaluate the leak before discharging the patient 12 days post-admission, which did demonstrate the fistula (Figure 5).

His pain became well controlled enough to be put on PO pain medications and eventually went on a regular diet via total parenteral nutrition with fluid restriction of 1.5 L/day for syndrome of inappropriate antidiuretic hormone secretion hyponatremia. He left against medical advice on day 16 of admission before the target end date of his antibiotic course.

The patient returned five days later complaining of worsening abdominal pain and stool leaking from outside his drains. Again, the surgery team did not want to perform any surgical intervention. They discussed the case with a gastroenterologist for endoscopic exploration. Flexible sigmoidoscopy was done, but the patient was deemed a poor candidate for endoscopic interventions. He was found to have a large sigmoid perforation. It was too large for a colonic stent to help with its closure, and there was also a high risk of stent migration. Without any other alternative solutions, the surgery team finally performed an exploratory laparotomy, and among their operation findings were two enterocutaneous fistulas in the right lower abdomen and one in the left lower abdomen with tracts to the stool collection in the pelvic through the sigmoid defect. There were also two colovesical fistulas - one in the anterior dome and one at the posterior fundus. There were two holes in the bladder - one larger anterior hole about 2 cm and another posterior hole about 0.5-1 cm. The patient had colovesicular fistulas takedown with bladder repair as well as enterocutaneous fistulas takedown. He also had a sigmoid resection with end colostomy. The patient stayed in the hospital until he was stable enough to be discharged.

## Discussion

The unusual connection between the bladder and colon, that of a colovesical fistula, is routinely diagnosed with a CT scan. A CT scan offers the benefit of identifying the location of the fistula, anatomic abnormalities, and possible malignancies [5]. However, this method of diagnosis with a CT scan has been shown to have varying sensitivity from 61% to 90% [4,8]. This diagnostic capability may be further complicated with smaller-sized fistulas. A CT scan is mentioned for the reasons above, but the diagnosis can still be made clinically as fecaluria and pneumaturia are pathognomonic signs of the pathology. Another study demonstrated successful surgical management of a patient with clinical signs but a lack of visibility on CT scans [9]. However, conservative management is also a viable option and has been shown to have similar outcomes compared to surgery [7]. In our study, a 65-year-old male presented with fecaluria and septic shock. The patient was deemed to be too unstable for surgery initially and was managed with antibiotics and intraperitoneal drainage. Since we suspected a colovesical fistula based on his clinical signs, we felt repeated CT scans were necessary to help us identify the fistula tract. The tracts were eventually identified and only when there were no alternative solutions and the patient became stable enough for surgery, he underwent successful takedown of the colovesical and enterocutaneous fistulas.

## Conclusions

Suspected colovesical fistula should be treated as such regardless of findings on CT. A CT scan may have a high but not perfect sensitivity for detection. Prompt treatment becomes important especially when measures need to be taken to prevent or remove infection caused by the fistula. With strong clinical suspicion, the colovesical fistula should be treated appropriately and with repeat CT scans to identify the fistula tract as needed. Surgery can be conducted depending on the stability of the candidate.
